# The Effect of Rhythmic Breathing on Pain of Dressing Change in Patients with Burns Referred to Ayatollah Mousavi Hospital

**Published:** 2018-01

**Authors:** Mehri Bozorg-Nejad, Hammed Azizkhani, Fatimah Mohaddes Ardebili, Sayed Kazem Mousavi, Farzad Manafi, Agha Fatemeh Hosseini

**Affiliations:** 1 Department of Medical-Surgical Nursing, Faculty of Nursing and Midwifery, Iran University of Medical Sciences, Tehran, Iran;; 2 Department of Surgery, Iran University of Medical Sciences, Tehran, Iran;; 3 School of Medicine, Iran University of Medical Sciences, Tehran, Iran;; 4 Department of Biostatistics, School of Public Health, Iran University of Medical Sciences, Tehran, Iran

**Keywords:** Rhythmic breathing, Pain, Burn, Dressing

## Abstract

**BACKGROUND:**

Burn is the worst tragedy among modern societies that individuals may experience. One of the most important problems of burns is pain; particularly at the time of treatment including burn dressings, debridement, surgical incisions and physiotherapy. The aim of this study was to determine the effect of rhythmic breathing on pain of dressing change in patients with burns.

**METHODS:**

This semi-experimental clinical trial study with a control group was conducted on 60 eligible burn patients who were selected using convenient sampling method and allocated randomly in two groups of test and control (each n=30). Data collection tools included demographic data and pain observation questionnaires. The rhythmic breathing was individually and orally trained to the patients of test group in a room separated by dividers for a 20-minute session. The pain intensity in test and control groups before and after dressing was investigated for three consecutive days.

**RESULTS:**

Friedman test results showed that pain intensity in both control and test groups had statistically significant differences. The pain intensity after rhythmic breathing reduced more in the test group, and this reduction was more significant during 3 days.

**CONCLUSION:**

Rhythmic breathing is an effective method on pain reduction of dressing change in patients with burn injuries.

## INTRODUCTION

Burn is among the worst tragedies in modern society that people may experience^1^ and it can occur in all age groups and social or economic classes.^2^ High frequency of severe burns and injuries results in mortality, morbidity and socioeconomic costs on society are good reason to pay special attention to burn victims by health experts and community as well.^3^ The World Health Organization announced that after the injuries caused by accidents, falls and violence, the injuries caused by burns are the world’s fourth most common injuries and burns prevalence in developing countries is 1.3 per 100,000 people; while these figure is 0.14 per 100,000 in developed countries. It is estimated that 270,000 deaths per year are caused by burns that the vast majority of them occurs in low- and middle-income countries.^4^

According to the latest studies in Iran, accidents, poisonings, suicides and fires have caused the highest fatality after circulatory diseases and the second cause of death after motor vehicle accidents is burns.^5^ The number of burning in Iran, with a population of 75 millions, is 150 thousand people annually, of which 25 to 30 thousand are hospitalized. Burn rate in our country was shown to be very high too.^6^ People suffering from burns also tolerate physical, psychological, and economic problems that pain is one of physical problems.^7^ Pain is so important that the American Pain Society has recognized pain as the fifth life sign in order to increase the importance of effective pain management among health care professionals.^8^

Among the large group of patients experiencing severe pain, there are people who have suffered from burns.^9^ The studies also have shown that acute levels of pain are related to the long-term negative psychological impacts such as depression, thoughts of suicide and post-traumatic stress syndrome for two years after the initial burn injury.^10^ Accordingly, decisive pain management during and after interventions such as physiotherapy and dressing is of particular importance.^11^ That’s why some healthcare centers use drugs as the standard treatment during this phase; unless there is a prohibition.^12^ Surprisingly, despite pharmacological interventions, 35% of people reported persistent pain, 75% pain interference with sleep, 56% interference with work, and 67% interference with their social function.^13^

Also, high doses of medications containing morphine increase the risk of complications such as respiratory failure, nausea, encephalopathy and constipation. These people also can show a degree of tolerance to painkillers.^14^ Since according to the gate control theory of Malzak and Wall, pain is not only a sensory experience, but also an emotional and cognitive experience.^15^ This theory encompasses physiological, psychological, cognitive and emotional aspects of pain and explains how to control pain by thoughts, feelings and behavioral practices and also directs the studies toward cognitive behavioral approaches to pain management.^8^

It is important to use non-pharmacological methods including alternative medicine to reduce patients’ discomfort and pain, in addition to pharmacological effects.^16^ Non-pharmacological interventions used to treat burn pain include hypnotism, art therapy and play therapy, distraction, music therapy and relaxation techniques.^17^ In the meantime, relaxation techniques are the most important non-pharmacological methods used for pain control.^17^ Relaxation by creating balance between the posterior and anterior hypothalamus, reducing the activity of the sympathetic system and catecholamine secretion can reduce muscle tension and physiological adverse effects such as lowering blood pressure, heart rate and muscle spasms caused by stress.^18^

Another advantage of relaxation in clinical environments is that the patients can apply it independently. Therefore, the use of relaxation in combination with painkillers provides a comprehensive approach of pain relief for patients. Relaxation is done in various ways, including the Progressive Muscular Relaxation (PMR), jaw relaxation, meditation, rhythmic breathing and so on.^16^ A simple relaxation technique is rhythmic abdominal breathing that is one of the simplest and most ancient relaxation techniques. In addition to relaxation advantages, this technique leads to cognitive distraction and a change in harmful stimulus structure like pain and stress.^19^

There is a variety of these techniques,^20^ such as abdominal breathing, variable breathing from the nasal passages, breathing against the resistance of respiratory tracts, breathing at different physical postures, holding breath. This technique can have palliative effects on anxiety, depression, stress disorders after accidents, chronic pain and stress caused by the disease.^21^ The aim of this study was to determine the effect of rhythmic breathing on pain of dressing change in patients with burns.

## MATERIALS AND METHODS

The present study is a semi-experimental clinical trial with a control group in which the effect of rhythmic breathing on pain of dressing change in people with burns was investigated. For doing so, researcher referred to Ayat Allah Mousavi Education and Treatment Center in Zanjan, Iran after getting certification from Ethics Committee and research certification. By the permission of Hospital Manager and Supervisor of Burn Ward and also introducing himself and explaining the objectives and procedure of the research, researcher invited the subjects who met inclusion criteria.

The inclusion criteria were the fourth day of hospitalization, daily dressing, older than 18 years, no inhalation and electric burns or self-immolation, burning level between 10% to 45% with some degree of 1, 2, 3, ability to communicate verbally and understanding Farsi, ability to read and write, ability to carry out co-breathing, lack of drug addiction, lack of mental illness, absence of severe hearing and vision problems. Sampling was in available way and subjects were enrolled after getting written consent. The patients were randomly divided into the test (n=30) and control (n=30) groups. To prevent bias, first the control group and then the intervention group entered the study.

Data collection tools included two questionnaires of demographic information and numeric pain scale. The scale included a 10 cm horizontal line graded based on the numerical pain scale from 1 to 10. Zero at this scale means no pain, 1 to 3 for mild pain, 4 to 6 for moderate pain, and 7 to 10 for severe pain. The subjects were asked to mark the point matched with their pain or tell its number to researcher. Pain Observation Scale was first designed in 1990 by Wewers and Lowe. This scale has been widely used in numerous empirical and clinical pain researches, and its validity and reliability are approved.^22^ This tool has been widely used to measure pain in Iran.^16^

At baseline, demographic data form was completed by the participants with the help of researcher. Then, in addition to routine care (including the use of drug before dressing), the rhythmic breathing method was individually and orally trained to the test group patients using in a private room separated by divisions. Training was done as follows: (i) Close your eyes, (ii) Relax the body muscles, (iii) Breath slowly and regularly and uniformly (Inhale, exhale, relax), so that breath in the air from your nose and then breathe out slowly through your mouth, and (iv) At all times of breathing, pay attention to the increased size of the chest, the movement of your abdomen and shoulders.

The training was carried out for at least 20 minutes, but training will continue to ensure learning and doing right by patient, and in the end, the participants were asked to complete this procedure for 20 times till the researcher will ensure the patient’s mastery by exact observation. After training the patients, they were asked to do this in practice in order to get mastery. And at the end, the test group was given a written guide. In addition, they were taught how to use the tools on the day of training. The control group received only routine care which contains painkiller medication at the beginning of dressing and it was conducted for patients in both groups depending on the severity of pain.

While the inclusion list of patients in the dressing room is available in the ward and arrangements have been made with the dressing room in this case, the pain numerical tool marked with code A was completed by the patient at the admission to the dressing room. Then the test group patients were asked to do rhythmic breathing during the dressing change that usually takes 10 minutes. Then a maximum of 5 minutes after leaving the dressing room, the pain numerical tool with code B was completed by the patient. This work was continued for 3 days. It is worth noting that the researcher was present at all stages of the study monitoring the patients. 

Ethical considerations After obtaining the required permit from the Ethics Committee of the university and officials of the selected hospital, data collection was carried out. In order to start sampling, the researcher introduced himself to the director of the burns unit of the hospital and identified the eligible subjects. Objectives and procedures of the study were explained to the participants, and informed consent was obtained from all the patients prior to participation. Moreover, participants were allowed to withdraw from the study at any time with no impact on their treatment process. After collecting the data and importing them into SPSS software (Version 20, Chicago, IL, USA), the data were analyzed using descriptive and inferential statistical methods. In connection with demographic characteristics, Chi-square, Wilcoxon and Mann-Whitney tests were used. A p value less than 0.05 was considered statistically significant.

## RESULTS

In connection with demographic characteristics, statistical anaysis showed that subjects in the two groups had no significant difference in terms of age (*p*=0.942), sex (*p*=0.436), education level (*p*=0.585), marital status (*p*=0.249), employment status (*p*=0.969), economic status (*p*=0.947), degree of burn (*p*=0. 154), cause of burn (*p*=0. 322), taking painkiller (*p*=0.739) and having an underlying illness (*p*=1); in other words, the two groups were homogeneous in terms of these characteristics. However, due to the homogeneity of demographic variables in the two groups, the results can be considered more reliably as a result of intervention.

According to Table 1, the mean age of patients in the control group was 32.66 years and the standard deviation was 8.73 years. Burned people in all groups often aged between 20 and 40 years old (76.7 percent) and the population of men and women was equal. The results of age frequency are due to the inclusion criteria where the subjects were over 18 years old. In terms of marital status, 46.7% of burned people were married. According to Table 1, the education level was as follows: 16.7% under high school diploma, 43.3% high school diploma, and 40% associate degree and above. According to the results of Table 1 in the control group, 10% of people had first-degree burn, 56.7% second-degree burn and 33.3% third-degree burn. 

**Table 1:** Comparison of control and test groups in terms of demographic variables.

**Variable**		**Control**		**Test**	
		**Number**	**Percent**	**Number**	**Percent**
Age	Under 25	6	20	7	23.3
	25-29	5	16.7	4	13.3
	30-34	5	16.7	5	16.7
	35-39	7	23.3	9	30
	40 and more	7	23.3	5	16.7
Gender	Woman	15	50	12	40
	Man	15	50	18	60
Education level	Under high school diploma	5	16.7	4	13.3
	High school diploma	13	43.3	8	26.6
	Associate degree	2	6.7	5	16.7
	BS	8	26.6	11	36.7
	BS and higher	2	6.7	2	6.7
Burns cause	Hot water and liquids	12	40	13	43.3
	Direct heat	15	50	16	53.4
	Chemicals	3	10	1	3.3
Place of burns	Leg	7	23.3	8	26.7
	Hand	26	86.7	20	66.7
	Body	8	26.7	11	36.3
	Face	8	26.7	10	33.3
	Back	2	6.7	2	6.7
Economic status	Very weak	11	36.7	9	30
	Weak	14	46.7	15	50
	Average	4	13.3	4	13.3
	Good	1	3.3	2	6.7

In the test group, over 70% had second- and third-degree burn. In terms of economic status, 95% of people were in moderate to low financial status. In connection with cause of burns, the results showed that the most frequent burns cause is direct heat with 52 percent of all people with burns. The other causes are boiling water and steam with 41% and chemicals with 7% of all causes of burns. In other resources, most of patients were burned by direct contact with flame or boiling water. Like this study, 95% of the cases were related to burning with direct flame.

Concerning the place of burns, 75% of the patients in this study burned their hands. After that, burning body and face constituted respectively 31.4 and 30% of people with burns (Table 1). The present study results showed that the mean pain change on the first day in the control group was 1.18±1.96 while the changes in the test group was 0.86±3.65; and the mean pain change on the second day in the control group was 1.38±1.69 while the changes in the test group was 0.77± 3.73; the mean pain change on the third day in the control group was 1.18±1.91 while the changes in the test group was 0.8±3.84.

Comparing the mean pain change score in the test and control group shows that both groups have experienced reduction in pain, but the pain changes in the test group was greater than the control. There was a significant difference in reduction of pain in the test group from the first to the third day, so that the mean reduction of pain in the test group increases over time. In other words, the test group subjects show a greater reduction of pain compared with the first day of rhythmic breathing (Table 2). With respect to the homogeneity of other items in the two groups, the changes can be seen as a result of intervention in the test group. In other words, it can be stated that rhythmic breathing reduces pain in the test group.

**Table 2:** Numerical indicators of pain changes in the patients of test and control groups.

**Group** **Pain Change**	**Control**	**Test**	**Mann-Whitney test result**
	**Mean±SD**	**Mean±SD**	
1^st^ day	1.91±1.52	3.65±0.86	*p*<0.001
2^nd^ day	1.69±1.38	3.73±0.77	*p*<0.001
3^rd^ day	1.96±1.18	3.84±0.80	*p*<0.001
Friedman test	*p*=0.16	*p*<0.001	

## DISCUSSION

In our study, in the test group, 70% of burns were second- and third-degree injuries, while 95% of patients were in moderate to low financial status. These findings have been reported before.^23^ Considering the cause of burns, the most frequent burn cause was direct heat among 52% of all patients with burn. The other causes were boiling water and steam with 41% and chemicals with 7% of all causes. In another report, most of patients were burned by direct contact with flame or boiling water. Like this study, 95% of the cases were related to burning with direct flame.^24^

Concerning the place of burns, 75% of the patients in this study had hand burns. After that, burning body and face constituted respectively 31.4 and 30% of people with burns, respectively. In another report, burning of hands had the highest frequency too.^25^ There was a significant difference in reduction of pain in the test group of our study, from the first to the third day, so that the mean reduction of pain in the test group increaseds over time. In other words, the test group subjects showed a greater reduction of pain compared with the first day of rhythmic breathing that is consistent with other studies in this area.^26^

The results showed that the test group had less pain intensity after dressing when compared with the control group and the rhythmic breathing was effective on pain intensity of burn dressing. The results are consistent with the results of previous studies including Park *et al*. who assessed the effect of breathing relaxation on burns dressing pain and anxiety^26^ and Gani *et al*. who evaluated the effect of breathing techniques on burns dressing pain: a randomized clinical trial^27^ and also Borzou *et al*. who studied the effect of rhythmic breathing on vascular needles pain intensity in patients undergoing hemodialysis.^28^

According to the results of this study, use of rhythmic breathing technique was effective as a non-pharmacological method to reduce pain of change dressing. According to gate control theory, it can be explained this way when the input from descending and inhibitory fibers of brain was more than stimulus input of small fibers, the gate is closed and the pain data cannot be Accordingly, relaxation can reduce or complete remove the pain through inhibitory impulses of the cerebral cortex and thalamus and thus closing the gate.

In addition, based on gate control theory, feeling out of control conditions can cause opening the gate through the cerebral cortex and increase different aspects of pain. As a result, burns patients need to feel they have control over their position. For this purpose, learning relaxation methods can create increasing feeling of personal control on pain and hence patients can play active role in learning and implementing pain management skills instead of being passive receiver of medical interventions. Relaxation reduces pain and consequently anxiety by releasing endorphins.^16^

Therefore, given the positive impact of rhythmic breathing on burns pain reduction and due to its easy learning, burned people can be encouraged to use this method when they feel suffering from pain and tension. Since nurses are mostly dealing with the phenomenon of dressing change pain among all professionals involved in the care of burns, and also because nurses are the initial defense of these people to reduce or relief of pain, it is suggested to train rhythmic breathing by nurses to the burned people so that patients may experience less pain before and after the dressing.

## References

[1] Mohtasham Amiri Z, Tanideh N, Seddighi A, Mokhtari M, Amini M, Shakouri Partovi A, Manafi A, Hashemi SS, Mehrabani D (2017). The effect of lithospermum officinale, silver sulfadiazine and alpha ointments in healing of burn wound injuries in rat. World J Plast Surg.

[2] Mohaddes F, Bozorg Nejad M, Manzari ZS (2016). Burn injury in Mottahari Burn Center in Tehran, Iran. World J Plast Surg.

[3] Duci BS, Arifi M H, Ahmeti R H, Zatriqi K V, Buja AZ, Hoxha TE, Mekaj YA (2015). Outcomes of older adults with burn injury: University Clinical Center of Kosovo. World J Plast Surg.

[4] (2012). World Health Statistics.

[5] Arshi S, Sadeghi-Bazargani H (2012). Mohammadi R. Burn injury-specific home safety assessment: a cross-sectional study in Iran. PLoSOne.

[6] Azizi A, Zarei J, Nabovati E, Vakili-Arki H, Abbasi E, Razavi AR (2014). Determining of the factors affecting mortality in burn patients using a decision tree data mining algorithm. Journal of Health Administration.

[7] Mohammadi AA, Tohidinik HR, Zardosht M, Seyed Jafari SM (2016). Self-burns in Fars Province, Southern Iran. World J Plast Surg.

[8] Smeltzer SC, Bare BG, Hinkle GL, Cheever KH (2012). Brunner and Suddarths Textbook of Medical Surgical Nursing.

[9] Najafi Ghezeljeh T, Mohades Ardebili F, Rafii F, Manafi F (2017). The effect of massage on anticipatory anxiety and procedural pain in patients with burn injury. World J Plats Surg.

[10] Wiechman SA, Sharar S, Mason ST, Patterson D (2009). Pain, pruritis and sleep following burn injury. Int J Psychiatry.

[11] Mohaddes F, Bozorg Nejad M, Manzari ZS (2014). Effect of educational program based on exercise therapy on burned hand function. World J Plats Surg.

[12] Brookman C, Kumar K, Wu CL, Honorio MD Benzon (2014). Burn Pain. Practical Management of Pain.

[13] Mohaddes Ardabili F, Purhajari S, Najafi Ghzeljeh T, Haghani H (2015). the effect of shiatsu massage on underlying anxiety in burn patients. World J Plast Surg.

[14] Hosseini Amiri M, Tavousi SH, Mazlom SR, Manzari ZS (2016). Effect of transcranial direct current stimulation on pain anxiety during burn wound care. Burns.

[15] elzack R, Katz J (2013). Pain. Wiley Interdiscip Rev Cogn Sci.

[16] Rafii F, Mohammadi-Fakhar F, Jamshidi OR (2013). Effectiveness of jaw relaxation for burn dressing pain: randomized clinical trial. Burns.

[17] LeMone P, Burke K, Dwyer T, Levett-Jones T, Moxham L, Reid-Searl K ( 2015). Medical-Surgical Nursing.

[18] Brown RP, Gerbarg PL, Muench F (2013). Breathing practices for treatment of psychiatric and stress-related medical conditions. Psychiatr Clin North Am.

[19] Kwekkeboom KL, Gretarsdottir E (2006). Systematic review of relaxation interventions for pain. J Nurs Scholarsh.

[20] Brown RP, Gerbarg PL (2005). Sudarshan kriya yogic breathing in the treatment of stress, anxiety, and depression: part II—clinical applications and guidelines. J Altern Complement Med.

[21] Seers K, Crichton N, Tutton L, Smith L, Saunders T (2008). Effectiveness of relaxation for postoperative pain and anxiety: randomized controlled trial. J Adv Nurs.

[22] Olf SE, Phelan HA, Arnoldo BD (2014). the year in burns 2013. Burns.

[23] Peck MD (2012). Epidemiology of burns throughout the World Part II: Intentional burns in adults. Burns.

[24] Al-Shaqsi SH, Al-Kashmiri A (2013). Al-Bulushi T Epidemiology of burns undergoing hospitalization to the national burns unit in the sultanate of oman: A 25-year review. Burns.

[25] Hsu KC, Chen LF, Hsiep PH (2016). Effect of music intervention on burn patients’ pain and anxiety during dressing changes. Burns.

[26] Park E, Oh H, Kim T (2013). The effects of relaxation breathing on procedural pain and anxiety during burn care. Burns.

[27] Gani H, Safar A, Safdari A (2013). The effect of breathing techniques on burn dressing pain: Randomized clinical trial. J Clin Nurs.

[28] Borzou SR, Akbari S, Falahynia GH, Mahjoub H (2013). The effect of rhythmic breathing on the vascular needles pain intensity in patients undergoing hemodialysis. Life Quarterly.

